# Genome Characteristics Reveal the Biocontrol Potential of Actinobacteria Isolated From Sugarcane Rhizosphere

**DOI:** 10.3389/fmicb.2021.797889

**Published:** 2021-12-23

**Authors:** Zhen Wang, Manoj Kumar Solanki, Zhuo-Xin Yu, Muhammad Anas, Deng-Feng Dong, Yong-Xiu Xing, Mukesh Kumar Malviya, Fei Pang, Yang-Rui Li

**Affiliations:** ^1^Guangxi Key Laboratory of Agricultural Resources Chemistry and Biotechnology, College of Biology and Pharmacy, Yulin Normal University, Yulin, China; ^2^Key Laboratory of Sugarcane Biotechnology and Genetic Improvement (Guangxi), Guangxi Key Laboratory of Sugarcane Genetic Improvement, Ministry of Agriculture, Sugarcane Research Institute of Guangxi Academy of Agricultural Sciences, Nanning, China; ^3^Agricultural College, Guangxi University, Nanning, China; ^4^Plant Cytogenetics and Molecular Biology Group, Faculty of Natural Sciences, Institute of Biology, Biotechnology and Environmental Protection, University of Silesia in Katowice, Katowice, Poland

**Keywords:** actinobacteria, sugarcane, genome, biocontrol, smut

## Abstract

To understand the beneficial interaction of sugarcane rhizosphere actinobacteria in promoting plant growth and managing plant diseases, this study investigated the potential role of sugarcane rhizospheric actinobacteria in promoting plant growth and antagonizing plant pathogens. We isolated 58 actinobacteria from the sugarcane rhizosphere, conducted plant growth-promoting (PGP) characteristics research, and tested the pathogenic fungi *in vitro*. Results showed that BTU6 (*Streptomyces griseorubiginosus*), the most representative strain, regulates plant defense enzyme activity and significantly enhances sugarcane smut resistance by regulating stress resistance-related enzyme (substances (POD, PAL, PPO, TP) in sugarcane) activity in sugarcane. The genomic evaluation indicated that BTU6 has the ability to biosynthesize chitinase, β-1,3-glucanase, and various secondary metabolites and plays an essential role in the growth of sugarcane plants under biotic stress. Potential mechanisms of the strain in improving the disease resistance of sugarcane plants and its potential in biodegrading exogenous chemicals were also revealed. This study showed the importance of sugarcane rhizosphere actinobacteria in microbial ecology and plant growth promotion.

## Introduction

The plant rhizosphere is a reservoir of microbial interactions that regulate plant activities. Plant roots exudates provide nutrients and energy substances to microbes (Fan et al., 2001; Bertin et al., 2003), and plant defense against pathogens in the rhizosphere by secreting antibiotics, inducing systemic resistance, or by direct antagonism against the pathogen for nutrient and space (Bakker et al., 2003); the roots also produce auxin (IAA), siderophores, dissolved inorganic phosphorus, which promote nutrient absorption and plant growth (Burdman et al., 2000).

Microorganisms are known to regulate plant gene expression, metabolism, growth and provide protection against multiple stress-causing factors (Ortíz Castro et al., 2009). Actinobacteria, which are gram-positive bacteria with high G + C DNA content that constitute one of the largest bacterial phyla, secrete several extracellular metabolites and antibiotics used as plant growth promoters (Strap, 2011). After biocontrol bacteria induce the host plant, it produces plant defense enzymes and disease-related proteins; lignin and phenol induction also directly improves the plant defense mechanisms (Zhu et al., 1995). A few studies have shown that *Streptomyces*, *Actinomadura*, *Micromonospora*, *Nocardia*, and other plant rhizosphere actinomycetes can enhance plant growth-promoting substances that increase plant biomass (Mishra et al., 1987; El-Tarabily et al., 2010). Moreover, several *Streptomyces* spp. have been recognized as potential biocontrol agents that indirectly increase plant disease resistance when pathogens invade the plant rhizosphere (Nimnoi et al., 2010). *Streptomyces* is widely utilized as a biocontrol agent due to its high sporulation capacity and the thick cell wall of its spores that result in long-term survival (Samac et al., 2003); this bacterium has a fast growth rate and is used as a bioinoculum in different forms, including powder, liquid, and cell biomass culture. For example, Mycostop, a biocontrol agent that contains actinomycetes cells, can control several soil-borne diseases such as *Rhizoctonia* spp., *Pythium* spp., *Fusarium* spp., *Phytophthora* spp. (Kortemaa et al., 1997; Hansen et al., 2010; Aggarwal et al., 2016), and Actinovate (containing 1% *Streptomyces lydicus* WYEC 108) and *Streptomyces griseoviridis* K61 have also been registered as biopesticides in the United States and France, respectively, and used in horticultural crops to manage soil-borne diseases, including *Fusarium* wilt and *Rhizoctonia* root rot (Yuan and Crawford, 1995; Minuto et al., 2006). In addition, *Streptomyces* secrete several antimicrobial compounds such as chitinase, glucanase, cellulase, organic acids, steroidal compounds, vitamins, and enzyme inhibitors (Mahadevan and Crawford, 1997; Palaniyandi et al., 2013). These metabolites plays an important role in plant diseases management and plant growth promotion (Olanrewaju and Babalola, 2019).

Plant diseases cause up to 25% of annual crop yield losses (Prashar et al., 2013). Plant growers well accept Microbe-based biological control technology in developed countries due to its low cost, environmental friendliness, and chemical-free residue. In recent years, microbes have been accepted as alternate sources for the biological control of plant diseases and plant growth promotion (Montesinos et al., 2002; Islam et al., 2016; Liu et al., 2018). Microbial biocontrol agents inhibit pathogens by different mechanisms, including antibiotics secretion, competition for food and space, direct parasitism, induced resistance of plant, and growth promotion of plant in stressed conditions (Van Wees et al., 2008; Kannan and Sureendar, 2009; de Jesus Sousa and Olivares, 2016; Viaene et al., 2016). However, the role of sugarcane rhizosphere actinomycetes in plant growth management and plant growth promotion is not well documented. In our previous studies, the actinobacterial genome has provided new insights on microorganisms that can improve drought resistance in plants (Wang et al., 2018). This study aimed to characterize actinomycetes in the sugarcane rhizosphere and their growth-promoting activity in order to provide new references for microorganisms that can antagonize plant pathogens and improve plant health.

## Materials and Methods

### Sample Collection and Bacterial Isolation

Rhizosphere soil samples were collected from experiment fields of Sugarcane Research Institute of Guangxi University, Nanning (Guangxi, China). The rhizosphere soil sampling was performed according to the procedure of Gobran and Clegg (1996). The sugarcane rhizosphere soil samples were collected from the root surface (0.5–5 mm) for the study. We used a modified method of Shahidi Bonjar et al. (2005) to isolate the actinomycetes. Briefly, 1 g of rhizosphere soil sample was added into a centrifuge tube containing 9 mL sterile water and incubated in a shaker at 100 rpm for 30 min. The suspension was subjected to gradient dilution and 200 μL of the diluted soil suspension was added to Gauze medium (Solarbio Biotech Co., Ltd., Beijing, China), containing 20 μg mL^–1^ nalidixic acid, and cultured at 30°C until colony appeared. Pure colonies were separately transferred into the Gauze agar plate. The purified strains were stored in 20% glycerol at –80°C. *Streptomyces chartreusis* WZS021 (Wang et al., 2016, 2018) was used as a reference strain for phylogenetic analysis and PGP characteristics.

### Plant Growth-Promoting Characteristics

To assess the production of indole acetic acid (IAA), 5 mL Luria broth was modified 0.5 mg mL^–1^ tryptophan, and the test strain was inoculated and incubated for 3 days on an incubator shaker at 180 rpm on 30°C. After incubation, the broth was centrifuged and to 1 mL of supernatant, 50 μL of 10 mmol L^–1^ orthophosphoric acid was added followed by the addition of 2 mL of Salkowski’s reagent for color development at 25°C incubation for 30 min in the dark; the optical density was taken at 530 nm. The different concentrations of IAA standard solution were used for the standard curve to quantify the IAA production (Gordon and Weber, 1951). For the siderophore test, the active strain was streaked onto the chrome azurol S (CAS) plate (Alexander and Zuberer, 1991) and incubated at 30°C. After 5 days, the presence or absence of an orange-yellow halo around the colonies on the medium was observed. The phosphate solubilizing ability of the strain was determined by inoculating the active strain in a phosphate dissolving medium (Hopebio Biological, Qingdao, China) and incubating for 5 days at 30°C; the halo area around the colony indicated that the bacteria had the ability to dissolve phosphorus. ACC deaminase activity was determined by measuring the production of α-ketobutyrate and ammonia produced by ACC cleavage according to (Penrose and Glick, 2003).

### DNA Extraction of Actinomycetes and Amplification of *16S* rRNA Sequences

Bacterial genomic DNA extraction kit (CWBIO Biotechnology Co., Ltd., Beijing, China) was used to isolate genomic DNA, and DNA purity and quantity were monitored using a NanoDrop One spectrophotometer (Thermo Fisher Scientific, Wilmington, DE, United States). PCR amplification of the *16S* rRNA and secondary metabolites synthase genes were carried out using the isolated genomic DNA of actinomycetes. The PCR system and reaction conditions are shown in Supplementary Tables 1, 2. The amplified PCR product was purified using the SanPrep column DNA gel recovery kit (Sangon Biotech Co., Ltd., Shanghai, China) and Sanger sequencing was performed in Sangon Biotech Co., Ltd. (Shanghai, China). The sequence reads in Fasta format were aligned in the NCBI database and accession numbers were obtained (Table 1). NCBI GenBank database using a BLASTn program. Identification to the species level was determined as maximum homology (C97%) to a type strain sequence in the GenBank. Evolutionary trees for the data sets were inferred by the neighbor-joining method of Saitou and Nei (1987) by using the neighbor-joining programby MEGA 6.0 software (Tamura et al., 2013) to construct the phylogenetic tree of actinomycetes.

**TABLE 1:** Identification and PGP characteristics of actinobacteria.

Isolates	Type strain match (% similarity)	No. of nucleotides	Accession number	PGP traits				
				^a^IAA	^b^ACC Deaminase	Siderophore	P-solibilization	N-free medium growth
TU2	*Nocardioides lutues* KCTC 9575^T^ (99.22)	1,416	MH497623	–	0.28^g^	+	–	+
TU3	*Streptomyces cinereoruber* NBRC 15396^T^ (98.45)	1,429	MH482864	–	–	+	–	–
TU4	*Streptomyces gramineus* JR043^T^ (97.99)	1,442	MH482865	21.89^m^	0.06^k^	–	–	–
TU5	*Streptomyces recifensis* NBRC 12813^T^ (98.17)	1,426	MH482866	–	0.26^g^	–	–	–
TU6	*Streptomyces lannensis* TA408^T^ (99.51)	1,424	MH482867	27.55^kl^	–	–	–	–
TU7	*Streptomyces griseoluteus* NBRC 13375^T^ (98.38)	1,424	MH482868	–	–	–	–	–
TU8	*Streptomyces filipinensis* NBRC 12860^T^ (98.60)	1,426	MH482869	25.83^l^	–	–	–	–
TU10	*Streptomyces bambusae* T110^T^ (98.95)	1,426	MH482870	–	–	–	+	+
TU11	*Streptomyces cyaneus* NRRL B02296^T^ (99.16)	1,427	MH482871	49.05^h^	–	–	+	–
TU12	*Streptomyces shaanxiensis* CCNWHQ 0031^T^ (98.73)	1,426	MH482872	43.75^i^	–	–	–	–
TU13	*Streptomyces neopeptinius* KNF 2047^T^ (98.71)	1,422	MH482873	75.55^c^	0.51^f^	+	–	–
TU14	*Streptomyces filipinensis* NBRC 12860^T^ (98.45)	1,423	MH482874	–	–	–	–	–
TU15	*Streptomyces pseudovenezuelae* DSM 40212^T^ (98.88)	1,430	MH482875	48.01^h^	0.01^k^	–	–	–
TU16	*Streptomyces griseorubiginosus* DSM 40469^T^ (99.02)	1,429	MH482876	58.67^de^	0.55^f^	–	–	–
TU17	*Streptomyces griseoluteus* NBRC 13375^T^ (98.10)	1,427	MH482877	–	–	–	–	–
TU19	*Streptomyces lannensis* TA408^T^ (99.30)	1,431	MH482878	–	–	–	–	+
TU20	*Amycolatopsis xylanica* CPCC 202699^T^ (99.15)	1,414	MH497624	36.02^j^	–	–	+	–
TU21	*Streptomyces griseoluteus* NBRC 13375^T^ (98.29)	1,408	MH482879	29.05^kl^	–	–	–	–
TU22	*Streptomyces recifensis* NBRC 12813^T^ (99.51)	1,425	MH482880	–	–	–	–	+
TU23	*Streptomyces lannensis* TA408^T^ (99.23)	1,424	MH482881	–	–	–	–	+
TU32	*Streptomyces pratensis* ch24^T^ (100)	1,427	MH482882	–	–	+	–	–
TU33	*Streptomyces neopeptinius* KNF 2047^T^ (98.85)	1,424	MH482883	40.71^i^	0.52^f^	–	–	–
BTU1	*Streptomyces shaanxiensis* CCNWHQ 0031^T^ (99.01)	1,423	MH482884	56.52^ef^	0.24^g^	+	+	–
BTU2	*Streptomyces neopeptinius* KNF 2047^T^ (98.93)	1,425	MH482885	60.96^d^	0.07^jk^	–	+	–
BTU3	*Streptomyces gramineus* JR043^T^ (97.99)	1,423	MH482886	–	–	–	–	+
BTU4	*Streptomyces griseoluteus* NBRC 13375^T^ (97.82)	1,423	MH482887	43.25^i^	–	–	–	–
BTU5	*Streptomyces recifensis* NBRC 12813^T^ (97.82)	1,426	MH482888	–	–	–	–	+
BTU6	*Streptomyces griseorubiginosus* DSM 40469^T^ (99.02)	1,428	MH482889	50.87^gh^	0.08^ijk^	+	–	+
BTU8	*Streptomyces amritsarensis* MTCC 11845^T^ (99.16)	1,428	MH482890	84.85^a^	–	+	+	–
BTU9	*Streptomyces pseudovenezuelae* DSM 40212^T^ (99.16)	1,423	MH482891	88.65^a^	1.47^d^	–	–	–
BTU10	*Streptomyces canus* DSM 40017^T^ (98.95)	1,426	MH482892	56.99^def^	0.15^hij^	–	–	–
BTU11	*Streptomyces pseudovenezuelae* DSM 40212^T^ (98.95)	1,426	MH482893	79.45^b^	0.16^hi^	–	–	–
BTU12	*Streptomyces neopeptinius* KNF 2047^T^ (98.93)	1,428	MH482894	54.30^fg^	0.07^jk^	–	–	+
BTU13	*Streptomyces amritsarensis* MTCC 11845^T^ (99.30)	1,424	MH482895	+	–	–	+	–
BTU14	*Streptomyces neopeptinius* KNF 2047^T^ (99.00)	1,427	MH482896	–	0.26^g^	–	+	–
BTU16	*Streptomyces cyaneus* NRRL B02296^T^ (98.94)	1,421	MH482897	59.89^de^	1.56^c^	–	–	–
BTU17	*Streptomyces pseudovenezuelae* DSM 40212^T^ (98.88)	1,426	MH482898	87.47^a^	0.08^h–k^	–	–	–
BTU18	*Streptomyces shaanxiensis* CCNWHQ 0031^T^ (99.01)	1,425	MH482899	51.16^gh^	0.08^ijk^	+	–	+
BTU19	*Streptomyces pratensis* ch24^T^ (100)	1,424	MH482900	–	0.76^e^	–	–	+
BTU20	*Streptomyces neopeptinius* KNF 2047^T^ (98.64)	1,424	MH482901	56.52^ef^	0.16^h^	–	+	+
BTU21	*Streptomyces gramineus* JR043^T^ (98.13)	1,429	MH482902	–	0.30^g^	–	–	–
BTU22	*Leifsonia soli* TG-S248^T^ (98.64)	1,425	MH497612	–	–	–	–	–
GEN1	*Streptomyces gramineus* JR043^T^ (97.99)	1,422	MH482903	–	–	–	–	–
GEN2	*Streptomyces violaceorubidus* LMG 20319^T^ (98.31)	1,423	MH482904	20.36^m^	–	–	–	–
GEN5	*Streptomyces glauciniger* CGMCC 4.1858^T^ (99.15)	1,420	MH482905	28.76^kl^	–	–	–	+
GEN7	*Streptomyces lannensis* TA408^T^ (99.79)	1,404	MH482906	21.89^m^	–	–	–	–
GEN8	*Streptomyces rhizophilus* JR041^T^ (99.15)	1,417	MH482907	35.20^j^	–	–	–	–
GEN15	*Streptomyces glauciniger* CGMCC 4.1858^T^ (99.29)	1,419	MH482908	–	–	–	–	+
WZS021	*Streptomyces chartreusis* NBRC 12753^T^ (99.63)	1,484	KX775948	30.50^k^	3.03^a^	+	–	+
WZS023	*Leucobacter aridicollis* CIP 108388^T^ (99.37)	1,427	MH497608	26.22^l^	–	+	+	–
WZS027	*Streptomyces badius* NRRL B02567^T^ (99.86)	1,403	MH482909	–	–	+	–	–
WZS028	*Streptomyces pratensis* ch24^T^ (99.78)	1,425	MH482910	–	–	–	+	–
WZS030	*Streptomyces pratensis* ch24^T^ (99.81)	1,429	MH482911	–	–	–	–	–
WZS031	*Streptomyces violascens* ISP 5183^T^ (99.93)	1,424	MH482912	–	–	+	–	–
WZS035	*Nocardioides lutues* KCTC 9575^T^ (100)	1,368	MH497610	21.32^m^	–	–	–	–
WZS050	*Streptomyces pratensis* ch24^T^ (100)	1,430	MH482913	–	–	–	+	–
WZS051	*Streptomyces pratensis* ch24^T^ (99.89)	1,428	MH482914	–	2.05^b^	+	–	–
WZS221	*Brevibacterium epidermidis* NBRC 14811^T^ (98.73)	1,424	MH559626	29.87^kl^	–	–	–	+

*+, positive; –, negative. ^a^mg⋅mL^–1^, SE: 2.07, DMRT (p = 0.05): 1.87, CV (%): 4.4; ^b^μmol α-ketobutyrate⋅mg^–1^ protein⋅h^–1^, SE: 0.09, DMRT (p = 0.05): 0.04, CV (%): 16.5. Different small letters as superscript represent significant difference at 95% confidence intervals based on DMRT test.*

### Detection of Antifungal Activity of Actinomycetes

To test the antagonism of actinomycetes, nine economically important plant pathogens were used in the dual culture assay (Supplementary Table 3). A 5 mm disc of full-grown pathogenic fungi was inoculated in the center of the potato dextrose agar plate, and an active actinomycetes strain was streaked 30 mm away from the disc of the pathogenic fungi. In each experiment, three replicates were performed, and pathogenic fungi without active strains were treated as controls. All plates were incubated at 28°C for 7 days, and mycelial growth and zone of inhibition were calculated as compared to control.

### Greenhouse-Based Biocontrol Assay Against the Smut Pathogen

The experiment was carried out in the sugarcane greenhouse located in the Guangxi University (Nanning, China). The indoor temperature was 24–32°C. The characteristics of the soil were as follows: pH, 6.76; organic matter, 21.42 g⋅kg^–1^; total nitrogen, 0.94 g⋅kg^–1^; total phosphorus, 2.73 g⋅kg^–1^; total potassium, 8.02 g⋅kg^–1^; alkaline nitrogen, 108 mg⋅kg^–1^; available phosphorus, 96 mg⋅kg^–1^; and available potassium, 79 mg⋅kg^–1^. We used three different treatments in this experiment: T1 (no inoculation, only water), T2 (inoculation of smut pathogen only), T3 (inoculation of smut pathogen and BTU6 as biocontrol agent). Thirty sugarcane plants were used per treatment and the experiment was repeated three times. Sugarcane stems with single shoots were soaked in water for 30 min at 52°C for hot water treatment, then soaked in a bacterial suspension (∼10^6^ cfu⋅mL^–1^) for 1 h at room temperature, followed by planting. A month later, T2 and T3 were treated with 100 mL of smut pathogen suspension (∼10^6^ cfu⋅mL^–1^) and T1 was treated with sterile water. After 2 months, the disease was analyzed and the leaves of three healthy sugarcane plants were collected for biochemical assays. Peroxidase (POD) activity was determined by the guaiacol method (Sakharov and Ardila, 1999). Phenylalanine ammonia-lyase (PAL) (Cat. No. BC0210), total phenol (TP) (Cat. No. BC1340), lignin (Cat. No. BC4200), and polyphenol oxidase (PPO) (Cat. No. BC0190) were determined using kits (Solarbio Biotech Co., Ltd., Beijing, China), according to the manufacturer’s instructions.

### Colonization of BTU6 in the Roots of Sugarcane

Sugarcane seedlings were maintained in plastic pots (20 cm in diameter, 18 cm in depth) filled with sterilized sand, which was moistened with sterile Hoagland nutrient solution, and kept in the greenhouse (28/22°C, 16/8 h light/dark cycle). BTU6 (10 mL, ∼10^6^ cfu⋅mL^–1^) was inoculated into a pot, and root samples were collected on day 14. After overnight fixation with 2.5% glutaraldehyde, the sample was dehydrated in a gradient series of acetone (30, 50, 70, 80, 90, and 100%; incubation of 20 min with each concentration of acetone). After drying with hexamethyldisilazane, the sample was coated with gold using an ion sputtering equipment (EM ACE200; Leica Microsystems, Wetzlar, Germany) and observed under a scanning electron microscope (SU8020; Hitachi High-Tech Instruments, Tokyo, Japan).

### Genome Sequencing of BTU6, Assembly, and Annotation

Cellular genome extraction is described with reference to the manufacturer’s protocol of bacterial genomic DNA extraction kit (CWBIO Biotechnology Co., Ltd., Beijing, China). The purity of extracted DNA was assayed with a NanoDrop One spectrophotometer (Thermo Fisher Scientific, Wilmington, DE, United States). The BTU6 genome was sequenced by Oxford Nanopore PromethION sequencing platform. Sequencing was carried out at the Biomarker Biotechnology Co., Ltd. (Beijing, China). After obtaining raw sequencing data, low-quality readings were filtered by SMRT 2.3.0 (Berlin et al., 2015; Koren and Phillippy, 2015) and the filtered sub-reads were assembled using Canu v1.5 software (Koren et al., 2017). Pilon software (Walker et al., 2014) was used to further correct the assembled genome, and a contig with higher accuracy and no gap was obtained.

We analyzed the Genome function using Gene Ontology (GO) (Ashburner et al., 2000), Kyoto Encyclopedia of Genes and Genomes (KEGG) (Kanehisa et al., 2004, 2006), Clusters of Orthologous Groups (COG) (Tatusov et al., 2003), Non-Redundant Protein Database databases (NR) (Li et al., 2002), Transporter Classification Database (TCDB) (Saier et al., 2014), Swiss-Prot (Bairoch and Apweiler, 2000), and TrEMBL (Magrane and Consortium, 2011). We analyzed and predicted the secondary metabolite synthetase genes of strain BTU6 using antiSMASH (Blin et al., 2019). The respected genomic sequence data was submitted to GenBank under the BioProject PRJNA549819 (accession number CP041168). Using the predicted genomic information, the application software Circos (Krzywinski et al., 2009) was used to draw a genomic circle map.

### Statistical Analysis

All data were analyzed using SPSS version 26 (IBM Corp., Armonk, NY, United States). One-way analysis of variance and Duncan’s multiple range test were used to determine differences between samples, with statistically significant differences set at a 5% level.

## Results

### Identification of Rhizosphere Actinomycetes

**FIGURE 1:**
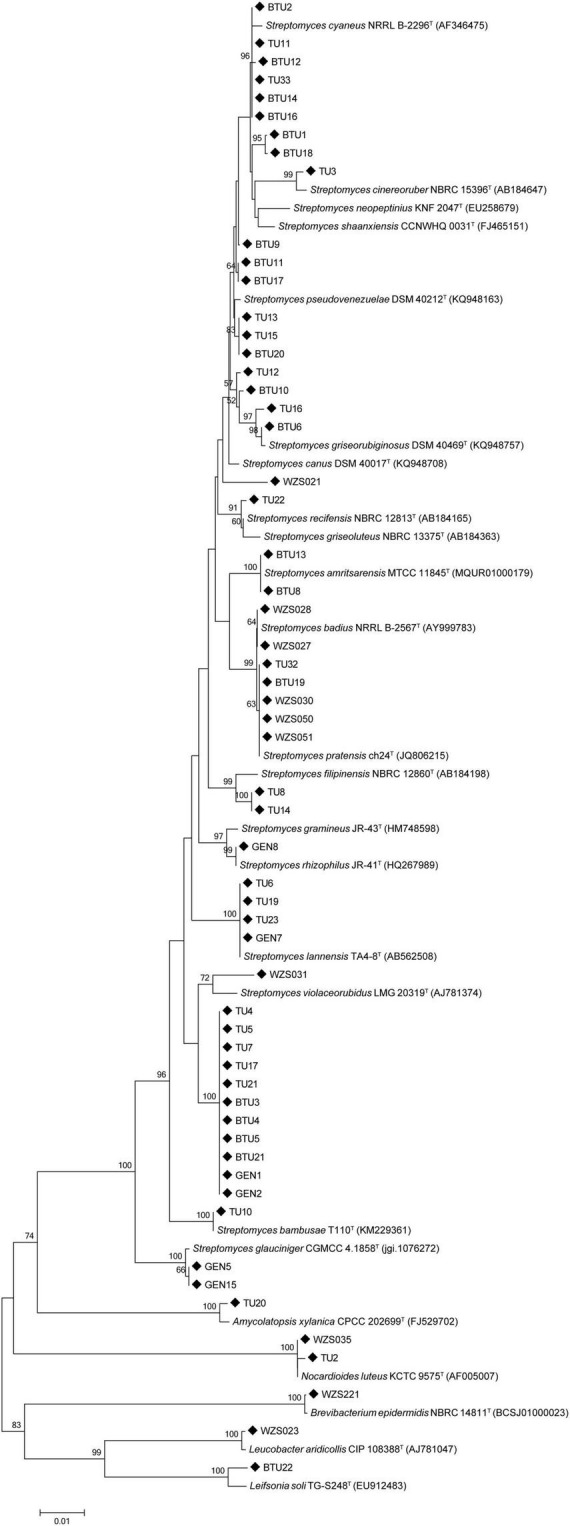
The phylogenetic tree was constructed using the 16S rDNA sequence of sugarcane rhizosphere actinomycetes and similar type strains through the neighbor-joining method.

We isolated a total of 58 actinobacteria from the sugarcane rhizosphere; of these, 52 strains were identified as *Streptomyces* (89%), and the remaining 6 non-*Streptomyces* actinomycetes strains were identified as *Leucobacter* (2%), *Nocardioides* (3%), *Leifsonia* (2%), *Amycolatopsis* (2%), and *Brevibacterium*(2%). Phylogenetic relationships among the isolates are shown in Figure 1.

### PGPR Characteristics of Actinomycetes

Among all isolates, 55% of strains had the ability to secrete IAA, with BTU9 showing the maximum IAA production of 88.65 mg mL^–1^. Moreover, 40% of active strains were able to secret ACC deaminase in the range 0.01–3.03 μmol α-ketobutyrate mg^–1^ protein h^–1^. The siderophores assay results indicated that 22% of strains had iron chelation ability. Among all strains, 21% of active strains had the ability to dissolve inorganic phosphorus (Table 1).

### Detection of Actinomycetes Activity Against Pathogenic Fungi in *in vitro*

**FIGURE 2:**
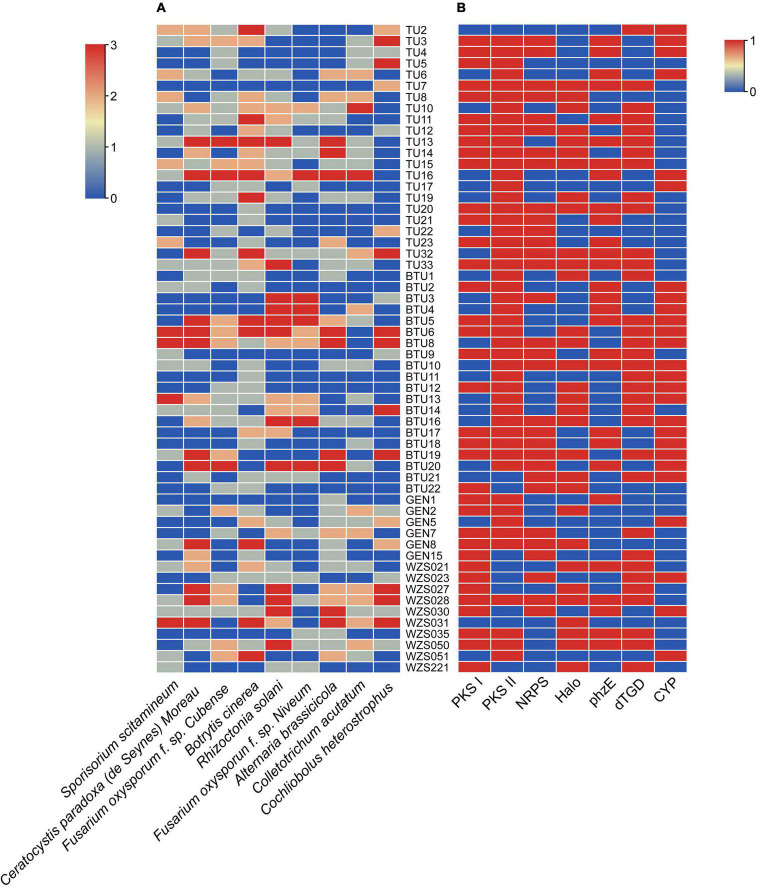
*In vitro* antagonism of actinobacteria and phytopathogenic fungi **(A)**, and screening of synthetic genes for secondary metabolites of actinobacteria **(B)**. **(A)** Growth definitely retarded with zone of inhibition (1) near the colony, (2) of ≥ 10 mm, and (3) of ≥ 15 mm. (0) Represents no inhibition. **(B)** (0) Negative; (1) positive.

The antagonism assays revealed that 72% actinomycetes showed antagonism against *Botrytis cinerea*, followed by 59% strains that antagonized against brown spot disease-causing *Alternaria brassicicola*, and 60% against the rice sheath blight-causing *Rhizoctonia solani* and banana wilt causing *Fusarium oxysporum* f. sp. *Cubense*. Among all strains, 37 actinomycetes antagonized more than four pathogenic fungi and only 2 strains showed antifungal activity against all tested pathogenic fungi (Figure 2A and Supplementary Table 3). However, some strains, including BTU6, BTU8, BTU13, and WZS031, had strong antagonistic effects on the *Sporisorium scitamineum*, and based on potential antagonistic ability, we used strain BTU6 for the greenhouse experiment.

### PCR Detection of Secondary Metabolite Synthase Genes

The secondary metabolite synthase gene detection test showed that the highest number (83%) of strains had *PKS II* gene, followed by *PKS I* gene (62%); the least number of strains (45%) had the *CYP* gene. Interestingly, 17 strains had five secondary metabolite synthase genes were commonly detected, and three secondary metabolite synthetase genes were widely detected in 51 strains (Figure 2B and Supplementary Table 4).

### Greenhouse Based Biocontrol Assay Against the Smut Pathogen

**FIGURE 3:**
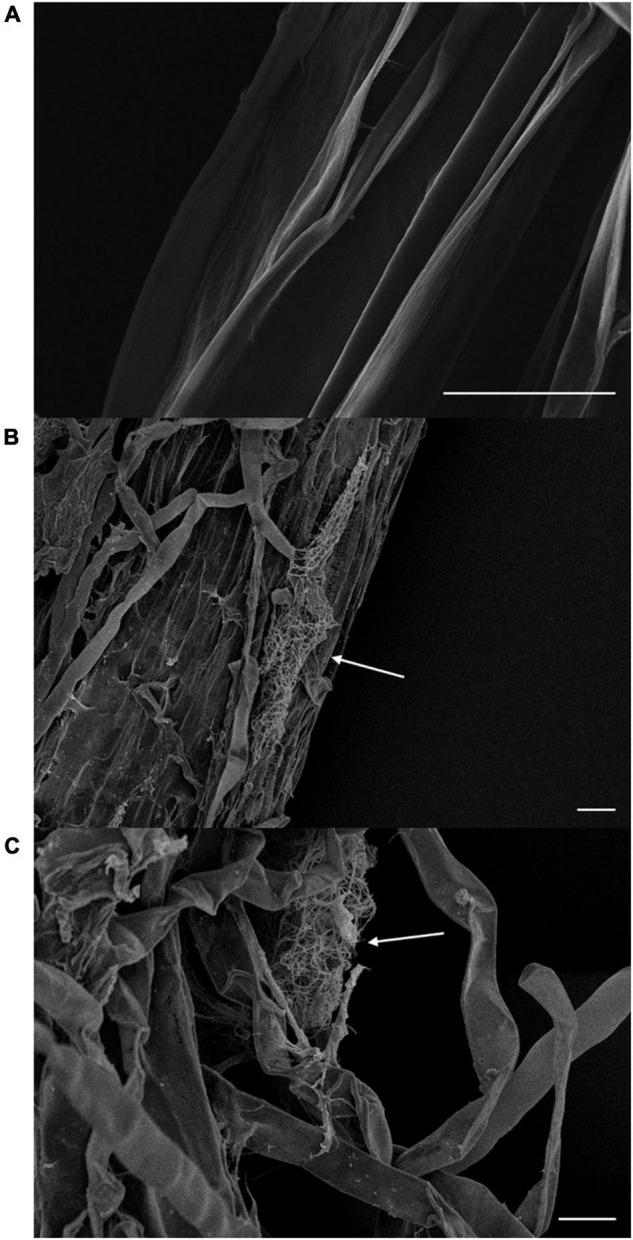
*Streptomyces griseorubiginosus* BTU6 colonizes the root surface **(B)** and hair area **(C)** of sugarcane and forms a mycelial cluster. **(A)** Represents a control sugarcane plant without BTU6 inoculation. Scale bar = 20 μm.

We assessed plant defense enzyme activity and disease-related substances, to examine the vital effect of BTU6 inoculation on plant antagonism against the sugarcane smut disease. The difference in POD, PPO, and PAL activity of the three treatments reached significant levels, with activity patterns in the respected treatments recorded as follows: T3 > T2 > T1. Compared with T1, The TP content in T2 and T3 increased by 54.5 and 121.2%, respectively. There was no significant difference in lignin in the leaves of inoculated and uninoculated plants (Table 2). The fresh weights of T2 and T3 were significantly higher than that of T1 (increased by 18.0 and 20.1%, respectively), but no significant difference in plant height was observed. Moreover, the presence of BTU6 enhanced the resistance of sugarcane to smut (Supplementary Figure 1). The smut infection rate of T3 was 12% higher than that of T1, but 10% lower than that of T2. The sterile sand inoculation test in the greenhouse also showed that BTU6 can colonize the root surface and root hair area of sugarcane (Figure 3).

**TABLE 2:** Comparison of defensive enzyme activities, active substances and susceptibility rates of plant disease resistance in different treatments in greenhouse experiments.

Treatment		POD (U⋅g^–1^)	PPO (U⋅g^–1^)	PAL (U⋅g^–1^)	TP (mg g^–1^)	Lignin (mg g^–1^)	Infection rate (%)	Fresh weight (g)		Plant height (m)
								Shoot	Root	
T1		90.1^c^	78.2^c^	26.9^c^	3.3^c^	153^a^	5.6^c^	154.9^b^	12.2^b^	0.62^a^
T2		105.9^b^	101.4^b^	57.4^b^	5.1^b^	161.5^a^	45.6^b^	174.4^a^	22.7^a^	0.67^a^
T3		138.6^a^	131.6^a^	67.4^a^	7.3^a^	157.9^a^	78.9^a^	182.1^a^	18.6^a^	0.63^a^
	SE	7.41	8.14	6.13	0.58	4.32	10.67	4.23	1.69	0.01
	LSD (*p* = 0.05)	5.57	7.19	1.79	0.22	11.70	3.51	3.49	2.0	0.02
	CV (%)	6.6	7.8	12.1	11.0	2.7	24.6	2.9	9.4	1.8

*T1 (no inoculation-only water), T2 (inoculation of smut pathogen only), T3 (inoculation of smut pathogen and actinomycetes BTU6 as biocontrol agent). Different small letters as superscript represent significant difference at 95% confidence intervals based on DMRT test.*

### Genomic Traits and Genes Associated With Antagonistic Diseases

**FIGURE 4:**
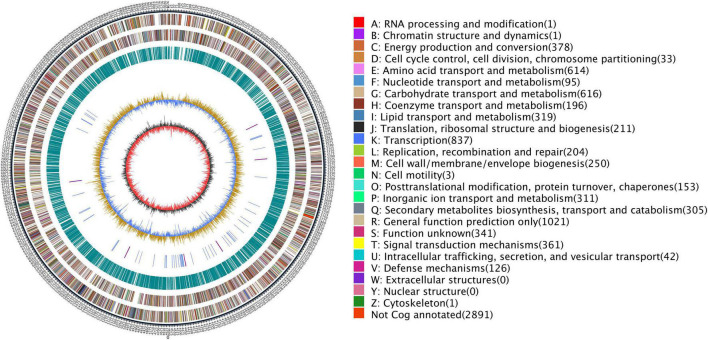
Genome overview map of *Streptomyces griseorubiginosus* BTU6. Circles from the outside to the inside represent the genome size, COG function classification, repetitive sequence, RNA classification, GC content, and GC-s.

**FIGURE 5:**
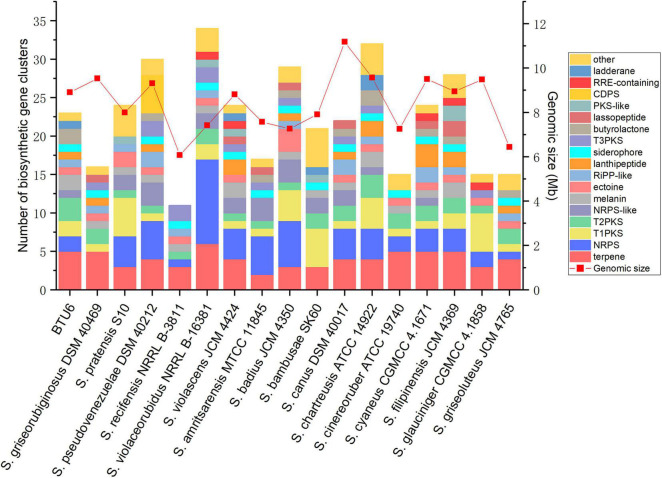
Comparison of 17 *Streptomyces* biosynthetic gene clusters and genome size, with similarity ≥ 20%.

After sequencing, the complete genome sequence of *Streptomyces griseorubiginosus* BTU6 was obtained. The genome contained a 9.22 Mb circular chromosome with a GC content of 71.15% (Figure 4 and Supplementary Table 5). Strain BTU6 contained 8,110 predicted protein-coding genes, 18S rRNA genes, 73 tRNA genes, and 42 ncRNA genes, related to the COG and GO functional categories (Supplementary Figures 2, 3). There were 305 genes involved in the biosynthesis, transport, and catabolism of secondary metabolites, 614 for amino acid transporters and metabolic genes, 319 for lipid transport and metabolic genes, 311 for inorganic ion transport and metabolism genes, and 616 for carbohydrate transport and metabolic genes. Combined with antiSMARSH results, strain BTU6 could produce a variety of peptidoglycans and polyketose-resistant compounds such as polyketide, t2pks, terpene, lanthipeptide, γ-butyrolactone (Supplementary Table 6). The synthesis of these secondary metabolites might help to inhibit the growth of the pathogen. A total of 34 biosynthetic gene clusters (BGCs) were identified in BTU6, of which 23 had a similarity more significant than 20%. The products encoded by these BGCs are the primary source of antibacterial activity of BTU6; of these products, atratumycin was demonstrated to be unique to BTU6. Upon a comparative analysis of the BTU6 and 16 *Streptomyces* genomes with similar genetic relationships, a total of 735 BGCs were predicted; the number of BGCs for a single strain ranged from 27 to 65. The differences in the number of BGCs among the strains were not significantly correlated with the genome size (*R*^2^ = 0.0898). According to the antiSMASH classification standard, these BGCs belonged to 38 categories, of which polyketide synthases (PKSs), terpenes, and non-ribosomal peptide synthetases (NRPSs) were the main types, accounting for a total of 306 (approximately 41.6%) of all BGCs (Figure 5).

## Discussion

Streptomyces are one of the most important actinobacteria genus associated with the plant rhizosphere and endosphere. The phylogenetic tree analysis showed that all *Streptomyces* were in the same clade, and other actinibacterial strains such as TU20, WZS035, and TU2, separated out in another clade. Strains WZS023 and BTU22 displayed a close relationship with each other.

Berg et al. (2006) reported that 35% of microorganisms isolated from the rhizosphere have the effect of inhibiting the growth of phytopathogenic microorganisms, while for results of microbial isolation by Fürnkranz et al. (2009), about two-thirds of the alternate microorganisms promoted plant growth. Hamdali et al. (2008) isolated 8 strains for dissolving inorganic phosphorus from mineral-containing phosphate soils in Morocco. The PGP properties screening results showed the strains with the highest IAA capacity were found (32, 55%), followed by the strains with ACC deaminase activity (23, 40%), and 89% of the strains had at least one kind of PGP ability. Strains indicating that sugarcane rhizosphere actinobacteria have the ability to promote plant growth.

Moreover, previous studies have discussed the secondary metabolite production of *Streptomyces*. Strobel (2003) reported that *Streptomyces* NRRL30562 strain produced four peptide antibiotics, Munumbicins A, B, C, and D, that could inhibit a variety of phytopathogenic bacteria, molds, and malaria parasites. Clermont et al. (2010) also revealed that geldanamycin produced by *Streptomyces melanosporofaciens* EF-76 had antagonistic effects on a variety of gram-positive bacteria and fungi. However, Yuan et al. (2014) confirmed that the PCR technology was a rapid method for detecting the secondary metabolite genes. In the present study, we also used PCR method to detect *PKS I*, *PKS II*, *NRPS*, *phz*E, *dTGD*, *Halo*, *CYP* genes. Interestingly, 83% strains had *the PKS II* gene (Supplementary Table 4) and 59% of strains had the *dTGD* gene. These results aligned with previous studies that reported on the important role of *dTGD* gene in the activity, toxicity, and solubility of natural products (Kirschning et al., 1997; Poulsen et al., 2000). D-glucose undergoes a series of complex actions, resulting in a structurally diverse 6-deoxyhexose, therefore, a very large family of 6-deoxyhexoses (Chen et al., 2011). From the highly conserved nature of the *dTGD* gene, the potential for the synthesis of 6-deoxyhexose by the *Streptomyces* strain was determined (Yu et al., 2004). Results showed that the *CYP* gene encoded a key enzyme in the biosynthesis of polyene antibiotics, cytochrome P450 hydroxylase (Lee et al., 2006). The halogenase gene in the molecule plays an important role in the biological activity of the entire compound. FADH_2_-dependent halogenase is the most important halogenase in the secondary metabolic halide biosynthesis pathway (Van Pee and Patallo, 2006), and the biosynthesis of many antibiotics is based mainly on FADH_2_-dependent halogenase (Van Pee, 2001). However, some strains have antibacterial activity, but no secondary metabolite genes have been detected; also, selected primers for amplifying metabolite genes have not been suitable for all actinobacteria due to differences in genotypes, although some of these genotypes could still be used as a basic understanding of biosynthetic genes. In the present study, the *PKS II* gene was detected in 83% of the strains, indicating that most of the sugarcane rhizosphere actinobacteria have the potential to synthesize type II polyketides. Even the smallest strain with the *CYP* gene was almost half the number of tested strains (26, 45%). Together with the PCR test of bioactive substance synthase, we also conducted an antagonistic test *in vitro*. All actinobacteria antagonized at least one pathogenic fungus, demonstrating the antagonistic properties of the actinobacteria.

Based on the *in vitro* results, we selected strain BTU6 for the sugarcane smut disease management experiment in the greenhouse, and results indicated that strain BTU6 increased plant disease resistance by regulating plant defense enzymes. Results on plant defense enzymes such as POD, PAL, and PPO in the BTU6 inoculated with smut pathogen showed that the activity levels were significantly higher (*p* < 0.05) than without BTU6, indicating that BTU6 inoculation enhanced the plant defense response against the smut pathogen. Ehret et al. (2010) also reported plant defense enzymes associated with disease resistance, of which POD, PAL, and PPO were essential enzymes involved in the plant protection process against the pathogen infection; these enzymes are often used as an important indicator of plant defense response (Lambais and Mehdy, 1995). Intermediates of PAL synthesis, such as phenolic substances and lignin are important antimicrobial substances in plants (Pellegrini et al., 1994). POD functions in strengthening plant cell walls, resisting the invasion of pathogens, and as a protective enzyme for scavenging reactive oxygen species in cells (Ray et al., 1998). PPO oxidizes phenolic substances into highly toxic terpenoids, thereby killing invading pathogens (Volpin et al., 1995). Lignin and its analogs are produced in large quantities when pathogens invade plants or are mechanically damaged (Vance et al., 1980; Hammerschmidt and Kuć, 1982; Nicholson and Hammerschmidt, 1992; Boudet et al., 1995). Lignified cell walls enhance the resistance of herbaceous and woody plants against pathogens (Hammerschmidt and Kuć, 1982; Southerton and Deverall, 1990; Dushnicky et al., 1998). Total phenolic content is also closely related to the disease resistance of plants. The total phenolic content of the cowpea varieties resistant to a mosaic disease was higher than the susceptible genotype (Shilpashree et al., 2013). Similarly, the content of phenolic substances in highly resistant red rot varieties was significantly higher than that in susceptible varieties. The change in total phenolic content could be used as a basis for assessing the degree of disease resistance of sugarcane (Beato et al., 1970). In this study, the total phenolic content of the three treatments was significantly different, indicating that phenolic substances also played a role in improving sugarcane smut resistance. Ippolito et al. (2000) found that *Aureobasidium pullulans* can significantly increase the activity of chitinase and β-1,3 glucanases in apple fruit, and degrade the fungal cell wall to inhibit fungal growth. In addition, Luo et al. (2012) found that the antagonistic yeast (*Pichia membranefaciens*) can induce the increase in POD activity in citrus fruits, and promote the formation of phenols and flavonoids, thereby improving the disease resistance of citrus. Zhao et al. (2008) studied the biocontrol effect of *Pichia guilliermondii* on tomato root rot (*Rhizopus nigricans*) and found that *Pichia guilliermondii* could induce the increase of SOD, CAT and POD activities in tomato fruit. This study also demonstrated that antagonistic bacteria could induce defense-related enzymes of sugarcane.

To explore the characteristics and commonalities between BTU6 and other *Streptomyces* strains, the BGCs of 17 *Streptomyces* strains were predicted and compared. Overall, different types of unknown gene clusters were identified in *Streptomyces*. PKS- and NRPS-related BGCs were the most prevalent; these two types of BGCs synthesize active substances of macrolides non-ribosomal peptides, respectively (Theobald et al., 2019). These observed differences between *Streptomyces* may be related to their complex living environment, thus representing adaptation events that they underwent. Moreover, these results confirmed the abundance of *Streptomyces*-derived naturally active substances in the sugarcane rhizosphere, which may be helpful for the development of new antibiotics. In particular, all *Streptomyces* were found to contain ectoine and siderophore BGCs. In the long-term evolutionary process, most microorganisms have developed an osmotic pressure mechanism that accumulates compatible solutes in the cytoplasm to counteract the changing external environment. Among them, ectoine is an important compatible solute (Fenizia et al., 2020), which enables *Streptomyces* to better adapt to the complex and changeable environment. Siderophores affect plant health by promoting plant nutrient absorption, improving plant resistance, and inhibiting the growth of pathogenic microbes (Verbon et al., 2017). In the present study, many heterozygous gene clusters were also identified in *Streptomyces*, thereby indicating that these bacteria exhibit a high degree of horizontal gene transfer in the long-term evolutionary process, which has essential ecological functions. Atratumycin is a cyclodepsipeptide that actively targets *Mycobacterium tuberculosis*. It is generally isolated from deep-sea *Streptomyces*, but it has also been reported in terrestrial *Streptomyces* (Sun et al., 2019). Considering the unique BGCs observed, BTU6 strains exhibit remarkable potential for application in the development of antimicrobial agents. Taken together, the common BGCs of *Streptomyces*, as well as the unique clusters identified, can explain the broad-spectrum antagonistic effect of BTU6 on common pathogens, mainly smut.

*In vivo* and *in vitro* experiments showed that BTU6 has the ability to enhance plant growth and also can improve plant defense against pathogens. These results motivated us to identify the genomic information to understand the plant growth promotion and stress regulation mechanisms. Interestingly, 34 secondary metabolic gene clusters in the genome of BTU6 concluded our results regarding the biocontrol potential. Several past studies have described the importance of chitinase to break down the chitinolytic cell wall of pathogens (Alexopoulos et al., 1996; Baharlouei et al., 2010; Chater et al., 2010; Shen et al., 2016). Polyketide compounds are one of the most diverse natural products of function and structure, with biological activities, including antibacterial, antiviral, antitumor, anti-pathogenic, anti-tuberculosis and immunosuppression (Miyanaga, 2017). NRPS can be used as an antibiotic, immunosuppressant, lipid-lowering and antifungal, and is widely used in medicine and agriculture (Misiek and Hoffmeister, 2007; Maansson et al., 2016). The metabolites of *Streptomyces* isolated from the soil also contain large amounts of terpenes (Wang C. et al., 2013; Wang Z. et al., 2013). Terpenoids not only have an antibacterial effect (Gallucci et al., 2009), but also have a strong killing effect on root-knot nematodes (Ntalli et al., 2011). Eisenman and Casadevall (2012) reported that melanin has anti-ultraviolet radiation and scavenging free radicals in organisms and can improve the ability of organisms to survive and compete. Lanthipeptide is a large class of cyclic peptide compounds containing thioether bonds (Cooper et al., 2010; Knerr and van der Donk, 2012) and has strong antibacterial activity against Gram-positive bacteria (Delves-Broughton et al., 1996). In this study, the defense enzyme activity of BTU6-treated sugarcane plants was different from that of untreated control plants and had a direct or indirect relationship with the expression of corresponding genes. *Streptomyces* is an essential class of biocontrol bacteria in agricultural production. Traditional experimental analysis and identification methods have limitations in analyzing *Streptomyces* active substances and cannot fully exploit their antibacterial potential; however, bioinformatics tools make up for these shortcomings. Bioinformatics provides an in-depth understanding of the nature and function of organisms from the perspective of the genome, and provides new approaches for microbial research (Bentley et al., 2002; Udwary et al., 2007; Kersten et al., 2011; Ziemert et al., 2012).

Persistent organic pollutants (POPs) have the characteristics of persistence, semi-volatility, easy bioaccumulation, and high toxicity, which bring great harm to human health and the environment (Lohmann et al., 2007; Lapworth et al., 2012; Tran et al., 2013). The microbial degradation method has received extensive attention due to its mild reaction conditions, thorough degradation, low operating costs, and no secondary pollution (Shokrollahzadeh et al., 2008; Hassan and Sorial, 2009; Rene et al., 2010). The genomic information generated from this study demonstrated the potential of BTU6 to biodegrade persistent organic pollutants. For example, hexachlorocyclohexanes (HCH) is the most common type of persistent organic pollutant. It was also the organic synthetic pesticide discovered and applied. Due to its excellent insecticidal effect, it is widely used in agriculture (Quintero et al., 2008). Benzoates, styrene, and caprolactam pose greater risks to environmental organisms (Marczynski et al., 2000; Dahlhoff et al., 2001; Willis and Ling, 2003). Bisphenol A (BPA) is often used as a compound to manufacture plastics, epoxies, and other materials. It is also an endocrine disruptor that interferes with the synthesis and metabolism of hormones and is associated with multiple pathologies in the human reproductive system (Howdeshell et al., 1999; Maffini et al., 2006; Diamanti-Kandarakis et al., 2009). Dioxins are often associated with waste incineration, which are carcinogenic, teratogenic, mutagenic, and are included in the first batch of Stockholm Convention on Persistent Organic Pollutants list (Everaert and Baeyens, 2002; Wang et al., 2012). The natural and synthetic polycyclic aromatic hydrocarbons are dispersed throughout the world with the flow of the atmosphere. Polycyclic aromatic hydrocarbons enter the vegetation from the atmosphere and eventually lead to enrichment in the food chain, which is the leading organic pollutant affecting human health (Blumer, 1976; Morehead et al., 1986). Atrazine was once considered one of the most difficult to degrade herbicides, posing a threat to aquatic ecosystems and human drinking water sources (Storrs and Kiesecker, 2004; Fan and Song, 2014). In addition, glycine oxidase in microorganisms degrades glyphosate to amine methylphosphonic acid (AM-PA) and glyoxylic acid to reduce glyphosate residues (Nishiya and Imanaka, 1998). BTU6 could degrade a variety of exogenous chemicals, especially those that are difficult to degrade by artificial methods, which provides a new solution to ecological protection.

## Conclusion

The sugarcane rhizosphere is rich in actinobacteria that promote plant growth, and most have the potential to antagonize plant pathogens. *Streptomyces griseorubiginosus* BTU6 enhances the resistance of plants to smut by regulating the stress resistance related enzyme activity and substances (POD, PAL, PPO, TP) in sugarcane. Bacterial genomic information also provides evidence that BTU6 produces a variety of secondary metabolites that antagonize fungi, further elucidating the molecular mechanisms of bacterial-plant interactions. In addition, the genome showed that BTU6 has the potential for biodegrading of a variety of exogenous chemicals, which can provide new insights into green solutions to various environmental options.

## Data Availability Statement

The datasets presented in this study can be found in online repositories. The names of the repository/repositories and accession number(s) can be found in the article/Supplementary Material.

## Author Contributions

ZW, Y-RL, D-FD, and FP designed the study. ZW, MS, and Z-XY conducted the experiments. MA and Y-XX analyzed the data. ZW, MS, Y-RL, and MM wrote the manuscript. Y-RL and D-FD revised and finalized the manuscript. All authors contributed to the article and approved the submitted version.

## Conflict of Interest

The authors declare that the research was conducted in the absence of any commercial or financial relationships that could be construed as a potential conflict of interest.

## Publisher’s Note

All claims expressed in this article are solely those of the authors and do not necessarily represent those of their affiliated organizations, or those of the publisher, the editors and the reviewers. Any product that may be evaluated in this article, or claim that may be made by its manufacturer, is not guaranteed or endorsed by the publisher.
