# Supplementary material for: Genome Characteristics Reveal the Biocontrol Potential of Actinobacteria Isolated From Sugarcane Rhizosphere
Source: Front Microbiol. 2021 Dec 23;12:797889. doi: 10.3389/fmicb.2021.797889 (PMC8740303; doi:10.3389/fmicb.2021.797889)
Supplement: Supplementary file 1 [file Data_Sheet_1.doc]

**Genome Characteristics Reveal the Biocontrol Potential of Actinobacteria isolated from Sugarcane Rhizosphere**

**Author information**

Zhen Wang^1,2,3†^, Manoj Kumar Solanki^4†^, Zhuo-Xin Yu^3^, Muhammad Anas^3^, Deng-Feng Dong^3^, Yong-Xiu Xing^3^, Mukesh Kumar Malviya^2^, Fei Pang^1*^, Yang-Rui Li^2,3*^

^1^College of Biology and Pharmacy, Guangxi Key Laboratory of Agricultural Resources Chemistry and Biotechnology, Yulin Normal University, Yulin, 537000, China

^2^Key Laboratory of Sugarcane Biotechnology and Genetic Improvement (Guangxi), Ministry of Agriculture, Guangxi Key Laboratory of Sugarcane Genetic Improvement, Sugarcane Research Institute of Guangxi Academy of Agricultural Sciences, Nanning, 530000, China.

^3^Agricultural College, Guangxi University, Nanning, 530000, China

^4^Plant Cytogenetics and Molecular Biology Group, Institute of Biology, Biotechnology and Environmental Protection, Faculty of Natural Sciences, University of Silesia in Katowice, 40‐032 Katowice, Poland

^†^ These authors have contributed equally to this work

^*^ Corresponding Author

**Running title:** Sugarcane actinobacteria as a biocontrol agent

Table S1 PCR reaction conditions for 16S rRNA gene and secondary metabolite synthesis genes

| Gene | Primer | Sequence (5’-3’) | Size (bp) | PCR conditions | References |
| --- | --- | --- | --- | --- | --- |
| 16S rRNA | 27F | AGAGTTTGATCCTGGCTCAG | 1400-1500 | Initial denaturation 94 °C for 5 min, 35 cycles of 94 °C for 55 s, 50 °C for 50 s, and 72 °C for 1 min, final extension 72 °C for 10 min | [Lane (1991)](#_ENREF_59) |
|  | 1492R | TACGGCTACCTTGTTACGACTT |  |  |  |
| PKS I | KSMA-F | TSGCSATGGACCCSCAGCAG | 700 | Initial denaturation 94 °C for 5 min, 35 cycles of 94 °C for 1 min, 60 °C for 1 min, and 72 °C for 2 min, final extension 72 °C for 5 min | [Izumikawa et al. (2003)](#_ENREF_47) |
|  | KSMB-R | CCSGTSCCGTGSGCCTCSAC |  |  |  |
| PKS II | 540F | GGITGCACSTCIGGIMTSGAC | 554 | Initial denaturation 94 °C for 5 min, 40 cycles of 94 °C for 1 min, 64 °C for 1 min, and 72 °C for 1.5 min, final extension 72 °C for 15 min | [Wawrik et al. (2005)](#_ENREF_121) |
|  | 1100R | CCGATSGCICCSAGIGAGTG |  |  |  |
| NRPS | A3F | GCSTACSYSATSTACACSTCSGG | 700 | Initial denaturation 95 °C for 5 min, 35 cycles of 95 °C for 30 s, 59 °C for 2 min, and 72 °C for 4 min, final extension 72 °C for 10 min | [Ayuso-Sacido and Genilloud (2005)](#_ENREF_5) |
|  | A7R | SASGTCVCCSGTSCGGTAS |  |  |  |
| Halo | FW | TTCCCSCGSTACCASATCGGSGAG | 500 | Initial denaturation 94 °C for 3 min, 30 cycles of 94 °C for 1 min, 58 °C for 90 s, and 72 °C for 1 min, final extension 72 °C for 5 min | [Hornung et al. (2010)](#_ENREF_43) |
|  | RV | GSGGGATSWMCCAGWACCASCC |  |  |  |
| *phz*E | phzEf | GAAGGCGCCAACTTCGTYATCAA | 450 | Initial denaturation 94 °C for 2 min, 35 cycles of 94 °C for 1 min, 55 °C for 1 min, and 72 °C for 2 min, final extension 72 °C for 6 min | [Schneemann et al. (2011)](#_ENREF_95) |
|  | phzEr | GCCYTCGATGAAGTACTCGGTGTG |  |  |  |
| dTGD | dTGD-1 | GSGGSGSSGCSGGSTTCATSGG | 600 | Initial denaturation 95 °C for 4 min, 40 cycles of 94 °C for 40 s, 50 °C for 40 s, and 72 °C for 90 s, final extension 72 °C for 5 min | [Du et al. (2004)](#_ENREF_27) |
|  | dTGD-2 | GGGWRCTGGYRSGGSCCGTAGTTG |  |  |  |
| CYP | PEH-1 | TGGATCGGCGACGACCGSVYCGT | 350 | Initial denaturation 94 °C for 5 min, 45 cycles of 94 °C for 1 min, 60 °C for 30 s, and 72 °C for 45 s, final extension 72 °C for 5 min | [Lee et al. (2006)](#_ENREF_61) |
|  | PEH-2 | CCGWASAGSAYSCCGTCGTACTT |  |  |  |

Lane, D.J. (1991) 16S/23S rRNA sequencing. In *Nucleic acid techniques in bacterial systematics*. Stackebrandt, E., and Goodfellow, M. (eds). Now York: John Wiley and Sons, pp. 115-175.

Izumikawa, M., Murata, M., Tachibana, K., Ebizuka, Y., and Fujii, I. (2003) Cloning of modular type I polyketide synthase genes from salinomycin producing strain of *streptomyces albus*. *Biorg Med Chem* **11**: 3401-3405.

Wawrik, B., Kerkhof, L., Zylstra, G.J., and Kukor, J.J. (2005) Identification of unique type II polyketide synthase genes in soil. *Appl Environ Microbiol* **71**: 2232-2238.

Ayuso-Sacido, A., and Genilloud, O. (2005) New PCR Primers for the Screening of NRPS and PKS-I Systems in Actinomycetes: Detection and Distribution of These Biosynthetic Gene Sequences in Major Taxonomic Groups. *Microb Ecol* **49**: 10-24.

Hornung, A., Bertazzo, M., Dziarnowski, A., Schneider, K., Welzel, K., Wohlert, S.E. et al. (2010) A genomic screening approach to the structure-guided identification of drug candidates from natural sources. *ChemBioChem* **8**: 757-766.

Schneemann, I., Wiese, J., Kunz, A.L., and Imhoff, J.F. (2011) Genetic approach for the fast discovery of phenazine producing bacteria. *Mar Drugs* **9**: 772-789.

Du, Y., Li, T., Wang, Y.G., and Xia, H. (2004) Identification and functional analysis of dTDP-Glucose-4,6-dehydratase gene and its linked gene cluster in an aminoglycoside antibiotics producer of *Streptomyces tenebrarius* H6. *Curr Microbiol* **49**: 99-107.

Lee, M.Y., Myeong, J.S., Park, H.J., Han, K., and Kim, E.S. (2006) Isolation and partial characterization of a cryptic polyene gene cluster in *Pseudonocardia autotrophica*. *J Ind Microbiol Biotechnol* **33**: 84-87.

Table S2 PCR reaction system for detecting secondary metabolite synthase genes

| Reaction component | Volume (μL) |
| --- | --- |
| 2×GoldStar MasterMix ^a^ | 12.5 |
| Template DNA (30 ng μL^-1^) | 1 |
| Primer-F | 1 |
| Primer-R | 1 |
| ddH2O | 9.5 |
| Total | 25 |

^a^ purchased from CWBIO Biotechnology (Beijing) Co., Ltd.

Table S3 Antagonism of actinobacteria and phytopathogenic fungi *in vitro*

| Isolates | Sugarcane smut disease | Sugarcane pineapple disease | Banana *Fusarium* wilt disease | Tomaoto gray mold | Rice sheath blight | Watermelon wilt disease | Black leaf spot of Chinese cabbage | Pepper anthracnose | Southern corn leaf blight |
| --- | --- | --- | --- | --- | --- | --- | --- | --- | --- |
|  | *Sporisorium scitamineum* | *Ceratocystis paradoxa* (de Seynes) *Moreau* | *Fusarium oxysporum* f. sp. *Cubense* | *Botrytis cinerea* | *Rhizoctonia solani* | *Fusarium oxysporun* f. sp. *Niveum* | *Alternaria brassicicola* | *Colletotrichum acutatum* | *Cochliobolus heterostrophus* |
| TU2 | ++ | ++ | + | +++ | + | - | - | - | ++ |
| TU3 | + | ++ | ++ | ++ | - | - | - | + | +++ |
| TU4 | - | - | + | - | - | - | - | + | + |
| TU5 | - | - | + | - | - | - | - | + | +++ |
| TU6 | ++ | + | - | + | + | - | ++ | ++ | - |
| TU7 | - | - | - | - | - | - | - | - | ++ |
| TU8 | ++ | - | + | ++ | + | - | ++ | ++ | - |
| TU10 | + | ++ | + | ++ | ++ | ++ | + | +++ | - |
| TU11 | - | + | + | +++ | ++ | + | + | - | - |
| TU12 | - | + | - | ++ | + | - | - | - | + |
| TU13 | + | +++ | +++ | +++ | +++ | + | +++ | + | - |
| TU14 | - | ++ | - | ++ | + | + | +++ | + | - |
| TU15 | ++ | + | ++ | ++ | + | - | + | + | - |
| TU16 | + | +++ | +++ | +++ | ++ | +++ | +++ | +++ | - |
| TU17 | - | - | + | + | - | + | - | - | - |
| TU19 | - | + | + | +++ | + | - | + | + | - |
| TU20 | - | - | - | + | - | - | - | - | - |
| TU21 | + | - | - | + | - | - | - | - | - |
| TU22 | - | - | + | - | + | - | - | - | ++ |
| TU23 | ++ | - | - | + | - | - | ++ | - | - |
| TU32 | - | +++ | + | +++ | + | + | + | ++ | +++ |
| TU33 | + | + | + | ++ | +++ | - | + | + | - |
| BTU1 | - | + | + | + | - | - | + | - | - |
| BTU2 | + | + | - | + | - | - | - | - | - |
| BTU3 | - | - | - | - | +++ | +++ | - | - | + |
| BTU4 | - | - | - | - | +++ | +++ | - | ++ | - |
| BTU5 | - | +++ | ++ | +++ | +++ | +++ | ++ | + | - |
| BTU6 | +++ | +++ | ++ | +++ | +++ | ++ | +++ | - | +++ |
| BTU8 | +++ | +++ | ++ | + | ++ | ++ | +++ | - | +++ |
| BTU9 | + | - | - | - | - | - | - | - | + |
| BTU10 | + | + | - | + | - | - | + | + | - |
| BTU11 | - | - | - | + | - | - | - | - | - |
| BTU12 | - | - | + | + | - | - | - | - | - |
| BTU13 | +++ | ++ | + | + | ++ | ++ | - | + | - |
| BTU14 | + | + | + | - | ++ | ++ | - | - | +++ |
| BTU16 | - | ++ | + | + | +++ | +++ | + | + | - |
| BTU17 | - | - | - | ++ | ++ | - | - | - | - |
| BTU18 | - | - | - | + | - | - | - | + | - |
| BTU19 | + | +++ | ++ | - | - | - | +++ | - | +++ |
| BTU20 | - | +++ | +++ | - | +++ | +++ | +++ | + | - |
| BTU21 | - | + | - | + | + | + | - | - | - |
| BTU22 | - | - | + | + | - | - | - | - | - |
| GEN1 | - | - | - | - | - | - | + | - | - |
| GEN2 | + | - | ++ | + | - | - | + | ++ | + |
| GEN5 | - | - | - | ++ | + | - | + | + | ++ |
| GEN7 | - | - | + | - | ++ | + | ++ | ++ | - |
| GEN8 | + | +++ | - | +++ | - | - | + | - | ++ |
| GEN15 | - | ++ | - | + | - | - | + | - | - |
| WZS021 | + | ++ | - | ++ | + | - | + | + | - |
| WZS023 | - | - | + | + | + | + | - | - | + |
| WZS027 | - | +++ | ++ | - | +++ | - | ++ | ++ | +++ |
| WZS028 | + | +++ | ++ | - | +++ | + | ++ | ++ | +++ |
| WZS030 | + | + | + | + | +++ | - | +++ | + | + |
| WZS031 | +++ | +++ | - | +++ | ++ | - | +++ | ++ | +++ |
| WZS035 | - | - | - | - | - | + | + | - | - |
| WZS050 | - | + | ++ | + | +++ | + | + | ++ | + |
| WZS051 | + | - | ++ | +++ | - | - | ++ | + | - |
| WZS221 | + | - | - | - | + | + | - | - | - |
| Total (Percentage of total isolates) | 31 (53%) | 33 (57%) | 34 (59%) | 42 (72%) | 35 (60%) | 23 (40%) | 34 (59%) | 30 (52%) | 23 (40%) |

Note: +, growth definitely retarded, with obvious zone of inhibition near colony; ++, with zone of inhibition of ≥10 mm; +++, with zone of inhibition of ≥15 mm; -, no inhibition.

Table S4 Screening of synthetic genes for secondary metabolites of actinobacteria

| Isolates | PKS I | PKS II | NRPS | Halo | *phz*E | dTGD | CYP |
| --- | --- | --- | --- | --- | --- | --- | --- |
| TU2 | - | - | - | - | - | + | + |
| TU3 | + | + | + | - | + | - | + |
| TU4 | + | + | + | - | + | - | + |
| TU5 | + | + | - | - | - | - | - |
| TU6 | - | + | - | - | + | - | + |
| TU7 | + | + | + | + | + | + | - |
| TU8 | + | + | + | + | - | - | - |
| TU10 | - | + | - | + | - | + | - |
| TU11 | + | + | + | - | + | + | - |
| TU12 | + | + | + | + | - | + | - |
| TU13 | + | + | - | + | + | + | - |
| TU14 | + | + | + | + | - | + | - |
| TU15 | + | + | + | + | + | + | - |
| TU16 | - | + | - | - | + | - | + |
| TU17 | - | + | - | - | - | - | + |
| TU19 | - | + | - | + | - | + | - |
| TU20 | + | + | + | + | + | + | - |
| TU21 | + | + | + | - | + | - | - |
| TU22 | - | + | + | - | - | - | - |
| TU23 | + | + | + | - | + | - | - |
| TU32 | - | + | + | + | + | + | - |
| TU33 | + | + | + | + | + | + | - |
| BTU1 | - | + | - | + | - | + | - |
| BTU2 | + | + | - | - | + | - | + |
| BTU3 | - | + | + | - | + | - | + |
| BTU4 | - | + | - | - | + | - | + |
| BTU5 | + | + | - | - | + | + | + |
| BTU6 | + | + | - | + | - | + | + |
| BTU8 | - | + | + | + | - | + | + |
| BTU9 | + | + | + | - | + | + | - |
| BTU10 | - | + | + | + | + | + | + |
| BTU11 | - | + | - | - | - | + | + |
| BTU12 | + | + | - | + | - | + | + |
| BTU13 | - | + | - | + | - | + | + |
| BTU14 | - | + | - | + | - | + | - |
| BTU16 | - | + | + | + | - | + | + |
| BTU17 | + | + | + | - | + | - | + |
| BTU18 | + | + | + | - | + | - | + |
| BTU19 | + | + | + | + | - | + | + |
| BTU20 | - | + | + | - | + | - | + |
| BTU21 | - | - | + | + | - | + | + |
| BTU22 | + | - | + | + | - | - | - |
| GEN1 | + | + | - | - | + | - | - |
| GEN2 | + | + | - | + | - | - | - |
| GEN5 | - | + | - | - | - | - | + |
| GEN7 | + | + | + | - | - | + | - |
| GEN8 | + | + | + | + | - | - | - |
| GEN15 | + | - | + | - | - | + | - |
| WZS021 | + | - | - | + | + | + | - |
| WZS023 | + | - | + | - | - | + | + |
| WZS027 | + | - | - | + | - | + | - |
| WZS028 | + | + | + | + | + | + | - |
| WZS030 | + | - | + | - | + | - | + |
| WZS031 | - | - | - | + | - | - | - |
| WZS035 | + | + | - | + | + | + | - |
| WZS050 | + | + | - | + | + | + | - |
| WZS051 | - | + | - | - | - | - | + |
| WZS221 | + | - | - | + | - | + | - |
| Total (Percentage of total isolates) | 36 (62%) | 48 (83%) | 31 (53%) | 31 (53%) | 28 (48%) | 34 (59%) | 26 (45%) |

Note: -, negative; +, positive.

Table S5 Genomic characteristics of actinobacterial strain *S. griseorubiginosus* BTU6

| Attribute | Value |
| --- | --- |
| Number of all scaffolds | 1 |
| Genome size (bp) | 9,226,027 |
| GC content (%) | 71.15 |
| rRNA | 18 |
| tRNA | 73 |
| other ncRNA | 42 |
| Protein-coding genes (CDS) | 8,110 |
| Pseudogene number | 5 |
| Secondary metabolite clusters | 34 |
| Genomic islands | 17 |
| Genes assigned to COGs | 5,219 |
| Genes assigned to GOs | 3,989 |
| Genes assigned to KEGGs | 2,734 |
| Proteins with signal peptides | 871 |
| Proteins with transmembrane helices | 1,955 |
| CRISPR | 117 |
| Prophage | 7 |
| Average coverage (×) | 243 |

Table S6 Prediction of the representative secondary metabolites in *S. griseorubiginosus* BTU6

| Type | Predicted production | Similarity (%) |
| --- | --- | --- |
| Thioamide-NRP | Lysocin | 9 |
| Melanin | Melanin | 71 |
| Terpene | Albaflavenone | 100 |
|  | Isorenieratene | 100 |
|  | Hopene | 92 |
|  | Foxicins A-D | 7 |
| T2PKS | Spore pigment | 83 |
|  | Julichrome | 62 |
|  | Granaticin | 37 |
|  | Methylenomycin A | 19 |
|  | Microansamycin | 64 |
|  | Akaeolide | 32 |
|  | Primycin | 8 |
| Siderophore | Desferrioxamin B/E | 83 |
| Ripp-like | Informatipeptin | 100 |
| NRPS | Kutzneride 2 | 24 |
| NAPAA | Teicoplanin | 3 |
| Ladderane | Atratumycin | 31 |
| Ectoine | Ectoine | 100 |
| CDPS | 9-methylstreptimidone | 19 |
| Butyrolactone | γ-butyrolactone | 100 |


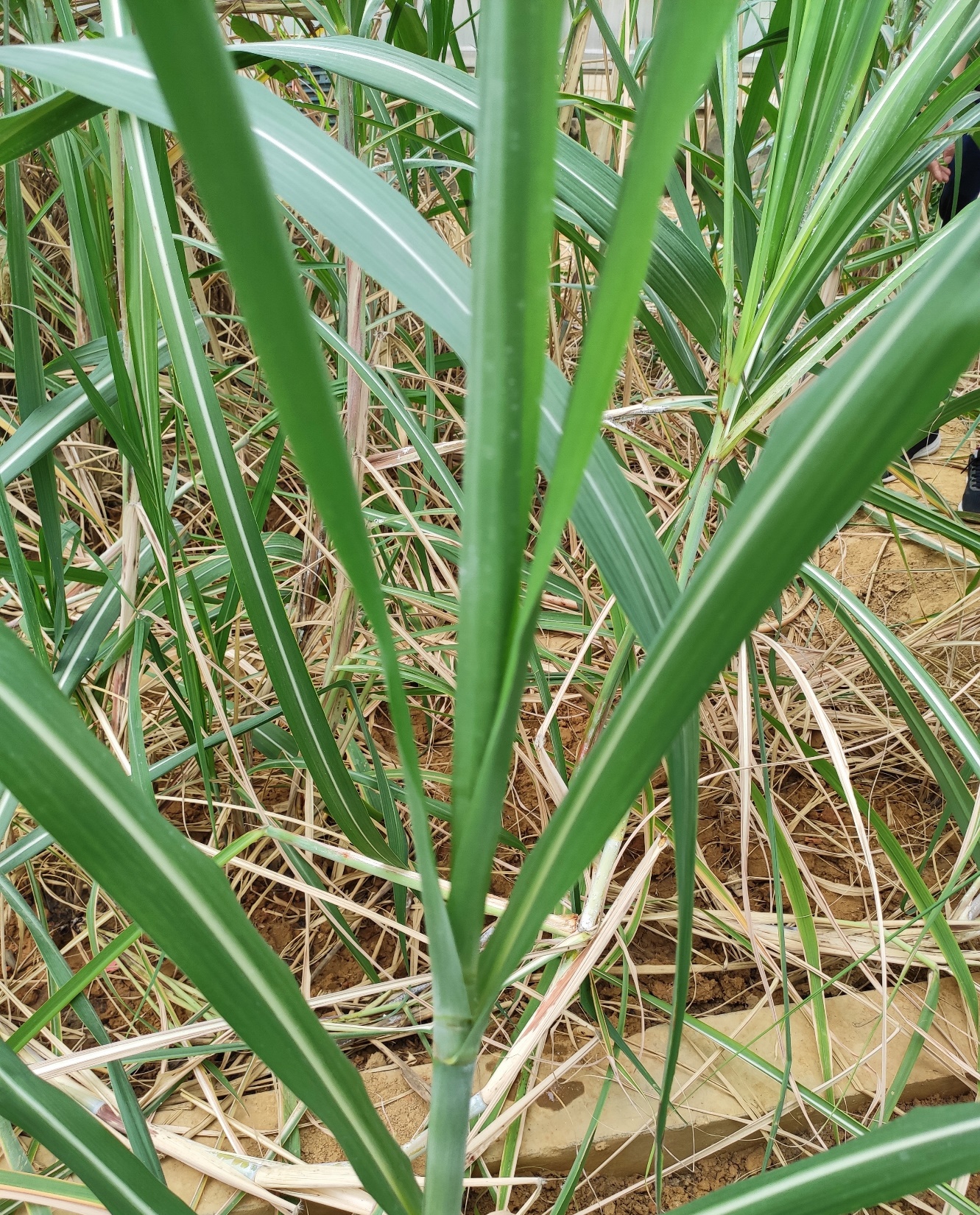

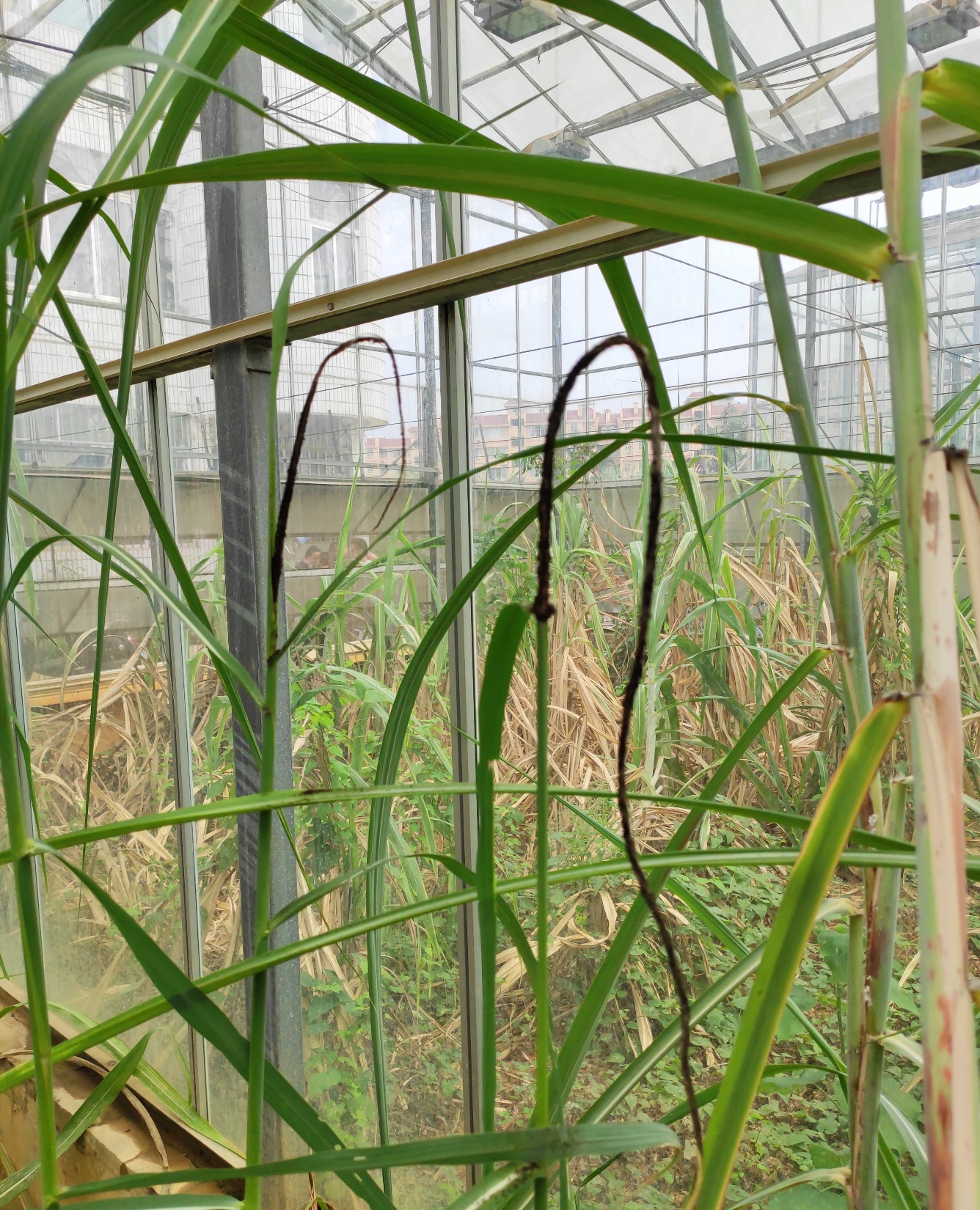


**a**

**b**

Figure S1 Biological control of sugarcane smut inoculated with *Streptomyces* BTU6 in the greenhouse. (a) Inoculation of smut pathogen and BTU6 as biocontrol agent, (b) inoculation of smut pathogen only


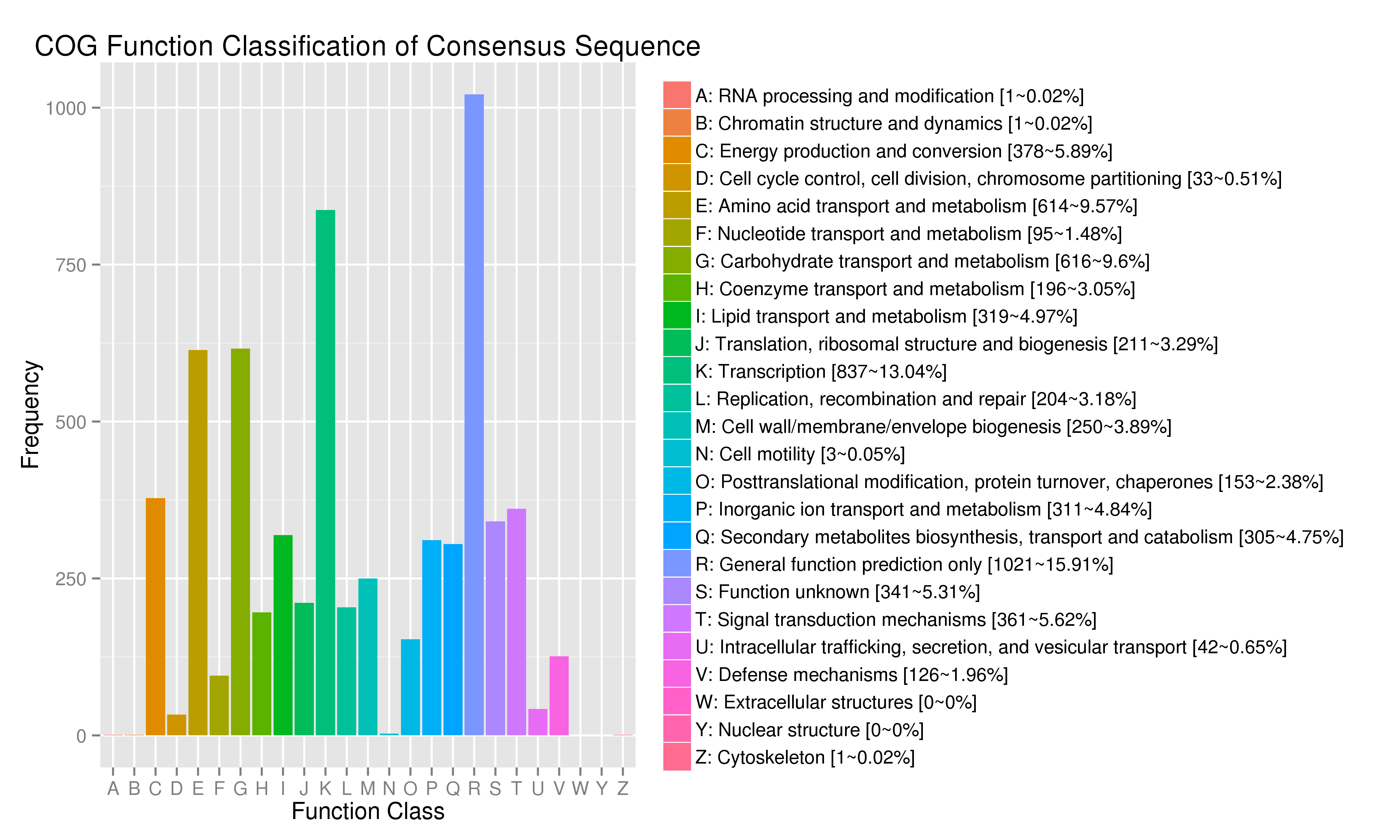
Figure S2 COG function classification predicted based on BTU6 genome sequence


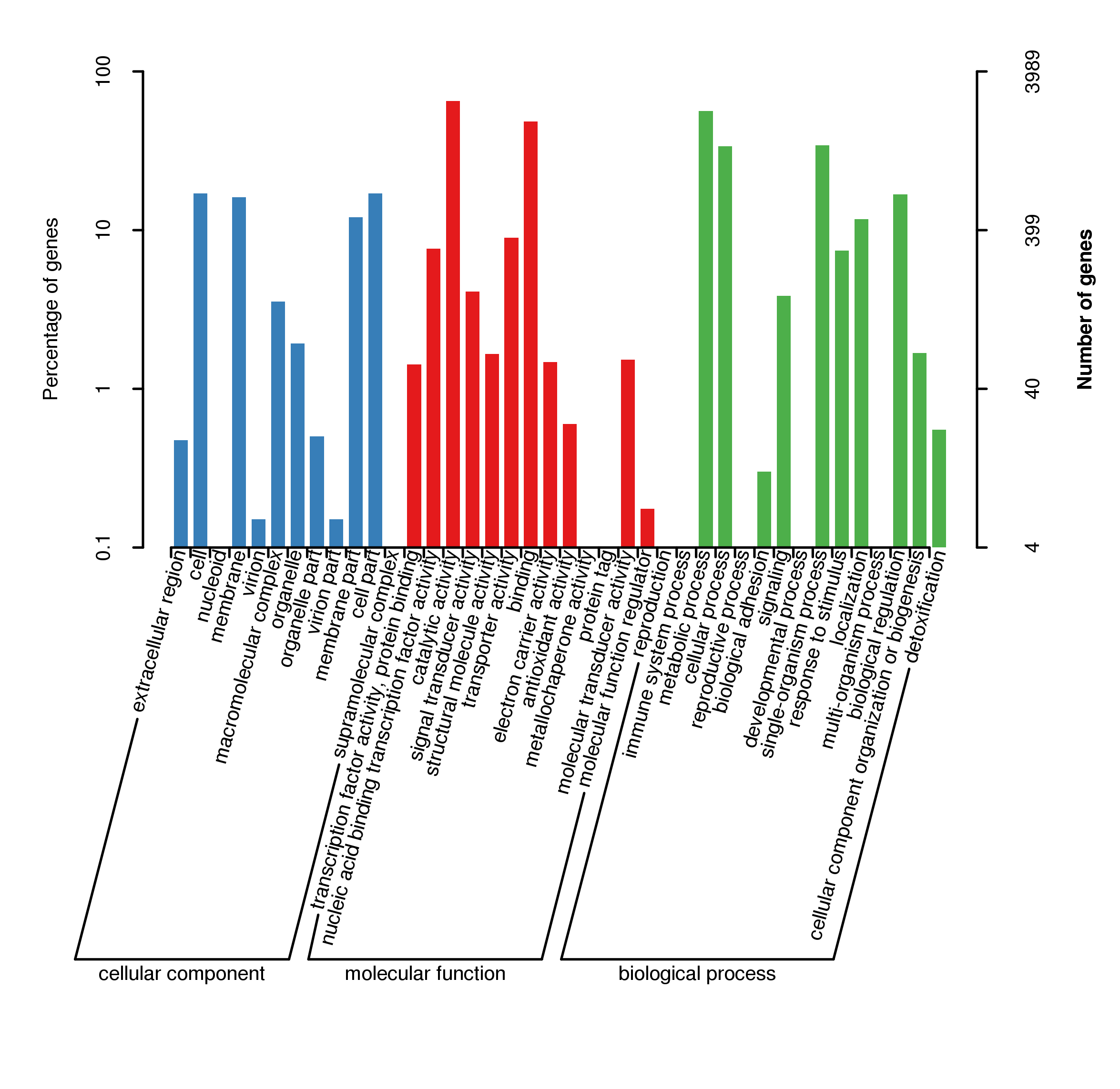


Figure S3 GO function classification predicted based on BTU6 genome sequence
